# Effects of parathyroid hormone and vitamin D supplementation on stroke among patients receiving peritoneal dialysis

**DOI:** 10.1186/s12882-020-01817-6

**Published:** 2020-05-18

**Authors:** Xiaohan You, Ying Zhou, Jianna Zhang, Qiongxiu Zhou, Yanling Shi, Zhen Su, Chaoshen Chen, Rongrong Shao, Ji Zhang

**Affiliations:** Department of Nephrology, the First Affiliated Hospital of Wenzhou Medical University, Nanbaixiang, Ouhai District, Wenzhou, 325000 Zhejiang P. R. China

**Keywords:** Continuous ambulatory peritoneal dialysis, Chronic kidney disease, Parathyroid hormone, Vitamin D, Stroke, Risk factor

## Abstract

**Background:**

Continuous ambulatory peritoneal dialysis (CAPD) patients have a high incidence of stroke and commonly have increased parathyroid hormone levels and vitamin D insufficiency. We seek to investigate the incidence of stroke and the role of parathyroid hormone and vitamin D supplementation in stroke risk among CAPD patients.

**Methods:**

This study employed a retrospective design. We enrolled a Chinese cohort of 980 CAPD patients who were routinely followed in our department. The demographic and clinical data were recorded at the time of initial CAPD and during follow-up. The included patients were separated into non-stroke and stroke groups. The effects of parathyroid hormone and vitamin D supplementation on stroke in CAPD patients was evaluated. The primary endpoint is defined as the first occurrence of stroke, and composite endpoint events are defined as death or switch to hemodialysis during follow-up.

**Results:**

A total of 757 eligible CAPD patients with a mean follow-up time of 54.7 (standard deviation, 33) months were included in the study. The median incidence of stroke among our CAPD patients was 18.9 (interquartile range, 15.7–22.1) per 1000 person-years. A significant nonlinear correlation between baseline iPTH and hazard of stroke (*p*-value of linear association = 0.2 and nonlinear association = 0.002) was observed in our univariate Cox regression analysis, and low baseline iPTH levels (≤150 pg/ml) were associated with an increased cumulative hazard of stroke. Multivariate Cox regression analysis indicated a significant interaction effect between age and iPTH after adjusting for other confounders. Vitamin D supplementation during follow-up was a predictive factor for stroke in our cohort.

**Conclusions:**

CAPD patients suffered a high risk of stroke, and lower iPTH levels were significantly correlated with an increased risk of stroke. Nevertheless, vitamin D supplementation may reduce the risk of stroke in these patients.

## Background

Continuous ambulatory peritoneal dialysis (CAPD) is a widely accepted and cost-effective therapy for patients with end-stage renal disease (ESRD) [1, 2], and the second or third cause of death among these patients is stroke [3]. The risk of stroke is increased greater than 3- to 10-fold among patients with ESRD than among the general population [4–6]. However, studies focused on risk factors for stroke among CAPD patients are rare.

According to previous studies, patients with ESRD share traditional risk factors for stroke with the general population [3, 7]. However, it is also recognized that the stroke risk profiles of CAPD patients are slightly different from those of the general population because of the loss of residual renal function, which may contribute to disorders of mineral and bone metabolism, volume overload, irrepressible hypertension, electronic disorder, and treatment with glucose-based dialysis solutions. Commonly, increased serum parathyroid hormone (PTH) levels and vitamin D insufficiency are universal among CAPD patients and are mostly attributed to chronic kidney disease-mineral and bone disorder (CKD-MBD) [8–10]. Accumulating evidence has indicated that vitamin D deficiency or insufficiency significantly increased cardiovascular disease (CVD) events among the general or dialysis population [11–14]. However, the relationship between serum PTH levels and CVD events is not consistent [15–18]. Studies have indicated that serum PTH levels may increase cardiovascular risk [15, 19]. However, a prospective study that recruited a cohort of 15,792 people in four U.S. communities with a median follow-up of 19 years failed to show that elevated PTH is an independent risk marker for incident cardiovascular disease [16]. Conversely, weak but significant inverse correlations (*p*-values = 0.02 to 0.04) of incident heart failure, peripheral artery disease, and CVD mortality with PTH were observed in this study, and these findings were also consistent with studies analyzing patients with dialysis [17, 20].

Few studies have focused on the relationship between stroke and CAPD, especially the role of PTH and vitamin D supplementation for stroke in patients with CAPD. Our study investigated the prevalence of stroke among patients with CAPD and the role of serum PTH levels and vitamin D supplementation in stroke risk via a retrospective study with a long-term, single-center follow-up.

## Methods

### Participants

This is a retrospective study based on a large cohort of CAPD patients that was conducted at a single center of the First Affiliate Hospital of Wenzhou University. A total of 1024 cases were identified and reviewed from our hospital information system and peritoneal dialysis database between Jan 2006 and Dec 2018. The inclusion criteria were as follows: 1. ESRD patients with CAPD and 2. routine follow-up for more than 3 months in our peritoneal dialysis center. The exclusion criteria were: 1. A history of continuous hemodialysis for more than 6 months prior to CAPD or a combination of continuous hemodialysis and CAPD, 2. a history of kidney transplantation, and 3. missing important laboratory data or medicine data. The study protocol was reviewed and approved by the Ethics Committee of the First Affiliated Hospital of Wenzhou University before the collection of any data, but additional informed consent was not obtained.

### Clinical data

At the initiation of CAPD, age, sex, blood pressure, serum creatinine, hemoglobin, albumin, intact parathyroid hormone (iPTH), uric acid, calcium, and phosphorus levels, and the history of diabetes, hypertension, chronic heart disease, atrial fibrillation, and stroke were recorded as baseline values. During follow-up, medications, including calcium channel blockers (CCBs), renin-angiotensin-aldosterone system (RAAS) blockers (angiotensin-converting enzyme inhibitors and angiotensin receptor blockers), vitamin D supplements (calcitriol and alfacalcidol), calcium agents, antiplatelet drugs, and anticoagulants, laboratory data and the first occurrence of stroke were recorded. The log-transformed, time-averaged and median absolute deviation (MAD) of iPTH were calculated for every case during follow-up. Patients who received medications for more than 3 months were assigned to the treatment group.

### Definitions

Stroke is defined as an episode of focal neurological deficit persisting for more than 24 h that are presumed to be caused by cerebral ischemia or hemorrhage. The diagnosis of stroke is verified by computer tomography or magnetic resonance imaging and evidence from the patients’ medical records. The primary endpoint is defined as the first-time occurrence of stroke, and composite endpoint events are defined as death or switch to hemodialysis during follow-up. Hypertension is defined as treatment with anti-hypertensive agents before peritoneal dialysis, a systolic blood pressure (SBP) ≥ 140 mmHg or a diastolic blood pressure (DBP) ≥ 90 mmHg after peritoneal dialysis catheterization in 1 month. Chronic heart disease is defined as evidence in medical records; treatment for coronary artery disease, arrhythmia, or congestive heart failure; or the presence of valvular heart disease. Patients lost to follow-up or those who did not reach an endpoint event during follow-up were censored. Survival time is defined from the initial time of CAPD until the date of the last follow-up at our peritoneal dialysis center or the time of occurrence of the endpoint event.

### Statistical analysis

Numerical data are expressed as the mean (standard deviation (SD)) for normally distributed data or median [interquartile range (IQR)] for skewed data, and categorical data are expressed as a count with a percentage (%). Included patients were divided into two groups based on the stroke status (non-stroke and stroke) to display the differences in clinical parameters, and examined using Student’s t-test or the Kruskal-Wallis test for numerical data and the chi-square test for categorical data. The difference in iPTH levels during the follow-up period between the non-stroke and stroke groups were determined using a nonlinear regression analysis fitted with a local polynomial regression equation. Then, Kaplan-Meier and Cox regression analyses were performed to calculate the cumulative risks of stroke and investigate the correlations of clinical parameters with stroke and composite endpoints. Considering the nonlinear relationship between the serum iPTH level and the risk of stroke, a spline term for the iPTH levels was built using the function *pspline* with two degrees of freedom from the *survival* package in R [21] to fit a nonlinear Cox regression model. Finally, the iPTH level was categorized into four groups (< 150, 150–300, 300–600, and > 600 pg/ml) according to the reference range provided in the Kidney Disease Improving Global Outcomes (KDIGO) [22] and the study by Morrone LF et al. [23]. A subgroup analysis was performed to assess the effects of vitamin D supplementation on stroke in different subgroups stratified by iPTH levels, age (≤ 65 and > 65 years), sex (male and female), serum calcium levels (≤ 2.1 and > 2.1 mmol/l), and serum phosphorus levels (≤ 1.5 and > 1.5 mmol/l). Plots were constructed and smoothed using the ggplot2 function in the R package [24]. All reported *p*-values are two-tailed, and p-values less than 0.05 are considered to indicate statistical significance. R (3.6.0, R Core Team) and R packages were used to process the data and perform statistical analyses [25].

## Results

**Fig. 1:**
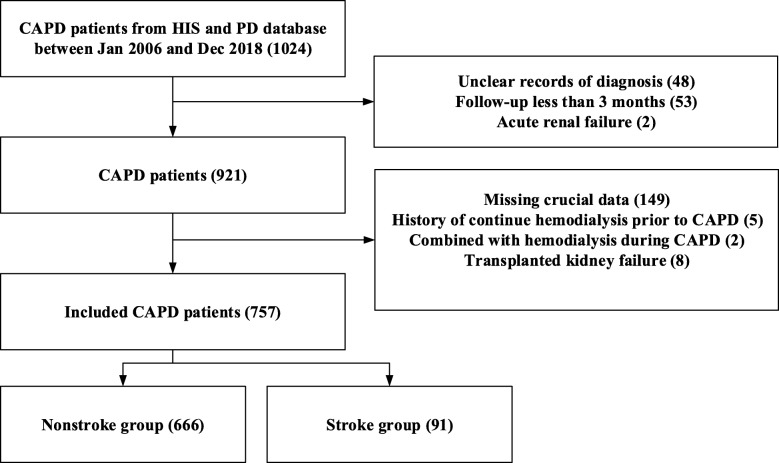
Flow chart of the patient inclusion process

A total of 757 eligible CAPD patients with a mean follow-up time of 54.7 (SD, 33) months were included in the study (Fig. 1). The median age of our cohort was 49 (IQR, 38–60) years, and the proportion of men was 55.1%. A total of 91 (12%) patients experienced stroke during a median follow-up time of 15 months and with a median occurrence age of 61.5 years and the counts of ischemic stroke and hemorrhagic stroke were 74 (83.1%) cases and 23 (25.8%) cases, with median ages of 64.5 and 55 years, respectively. The median incidence of stroke among our CAPD patients was 18.9 (IQR, 15.7–22.1) per 1000 person-years. Notably, patients had a higher incidence of stroke at CAPD initiation and 5 years and 10 years after CAPD (Supplemental Fig. 1). One hundred fifty-three (20%) patients in our cohort experienced composite endpoints, and the proportion of composite endpoint events increased significantly in the stroke group compared to the non-stroke group (39.6% vs 17.6%, respectively; *p*-value < 0.001).

A few significant differences were observed at the initiation of CAPD between the stroke and non-stroke groups in our cohort. The median age of the stroke group was significantly older (62 vs 48 years; *p*-value < 0.001), and the stroke group had lower serum albumin (33.6 vs 35.7 g/l, *p*-value = 0.002), serum phosphorus (1.6 vs 1.7 mmol/l, *p*-value = 0.001), iPTH (167.7 vs 269.0 pg/ml, *p*-value = 0.001) and DBP (76.6 vs 84.6 mmHg, p-value < 0.001) levels. Furthermore, the prevalence of chronic heart disease (97.8% vs 25.8%, p-value < 0.001) and diabetes (53.8% vs 24.3%, *p*-value < 0.001) was significantly higher in the stroke group. Interestingly, the prevalence of vitamin D supplementation was significantly lower in the stroke group than in the non-stroke group (53.8% vs 70.9%, respectively; p-value = 0.002). The results of the comparison between the stroke and non-stroke groups are shown in Table 1.

**Table 1:** Comparing the clinical characteristics and laboratory measurements of the included CAPD patients with and without stroke

Characteristics	Overall	Case-control		***p***-value
		Non-stroke	Stroke	
Patients (n)	757	666	91	
Male (n, %)	417 (55.1)	368 (55.3)	49 (53.8)	0.9
Age (years, median [IQR])	49.0 [38.0, 60.0]	48.0 [37.0, 58.0]	62.0 [52.5, 68.5]	< 0.001
Serum albumin (g/l, median [IQR])	35.4 [31.8, 38.9]	35.7 [32.0, 39.2]	33.6 [31.2, 37.2]	0.004
Hemoglobin (g/l, median [IQR])	81.1 (18.6)	81.3 (18.6)	80.0 (19.2)	0.5
Serum calcium (mmol/l, median [IQR])	2.1 [1.9, 2.2]	2.1 [1.9, 2.2]	2.1 [1.9, 2.2]	0.7
Serum phosphorus (mmol/l, median [IQR])	1.7 [1.5, 2.0]	1.7 [1.5, 2.0]	1.6 [1.3, 1.9]	0.002
iPTH (pg/ml, median [IQR])	258.9 [141.3, 416.5]	270.2 [153.9, 420.9]	167.7 [87.7, 346.2]	0.001
iPTH levels (n, %)				< 0.001
≤ 150	201 (26.8)	158 (24.0)	43 (47.3)	
150–300	235 (31.3)	215 (32.6)	20 (22.0)	
300–600	241 (32.1)	223 (33.8)	18 (19.8)	
> 600	73 (9.7)	63 (9.6)	10 (11.0)	
SBP (mmHg, mean (SD))	140.8 (25.1)	140.5 (24.9)	143.6 (26.6)	0.3
DBP (mmHg, mean (SD))	84.0 [73.0, 94.0]	85.0 [74.0, 94.0]	77.0 [66.0, 86.2]	< 0.001
Pulse pressure (mmHg, mean (SD))	57.4 (19.9)	56.2 (19.1)	67.4 (22.1)	< 0.001
**Comorbid diseases**				
Heart disease (n, %)	208 (27.5)	164 (24.6)	44 (48.4)	< 0.001
Atrial fibrillation (n, %)	27 (3.6)	21 (3.2)	6 (6.6)	0.2
Hypertension (n, %)	736 (97.2)	645 (96.8)	91 (100.0)	0.2
Diabetes (n, %)	211 (27.9)	162 (24.3)	49 (53.8)	< 0.001
**Agents**				
Antiplatelet drugs (n, %)	270 (35.7)	208 (31.2)	62 (68.1)	< 0.001
Anticoagulants (n, %)	15 (2.0)	14 (2.1)	1 (1.1)	0.8
RAAS blockades (n, %)	600 (79.3)	529 (79.4)	71 (78.0)	0.9
CCBs (n, %)	685 (90.5)	597 (89.6)	88 (96.7)	0.05
Calcium agents (n, %)	604 (79.8)	538 (80.8)	66 (72.5)	0.09
Vitamin D (n, %)	521 (68.8)	472 (70.9)	49 (53.8)	0.002
Statins (n, %)	466 (61.6)	399 (59.9)	67 (73.6)	0.02
Follow-up time (months, median [IQR])	54.7 (33.0)	54.6 (32.7)	55.5 (35.4)	0.8
Composite endpoint (n, %)	153 (20.2)	117 (17.6)	36 (39.6)	< 0.001

### Difference in iPTH levels during follow-up between the stroke and non-stroke groups

Compared to the non-stroke group, the median values of the original, log-transformed and time-averaged iPTH levels during follow-up decreased significantly in the stroke group (*p*-values = 0.001, < 0.001 and 0.001, respectively). Furthermore, the median absolute difference in serum iPTH levels also decreased significantly in the stroke group (52.9 [34.4, 93.2] vs 66.2 [41.7, 106.5], p-value = 0.03), indicating that the serum iPTH level was significantly reduced in the stroke group compared with the non-stroke group during follow-up (Supplemental Table 1).

**Fig. 2:**
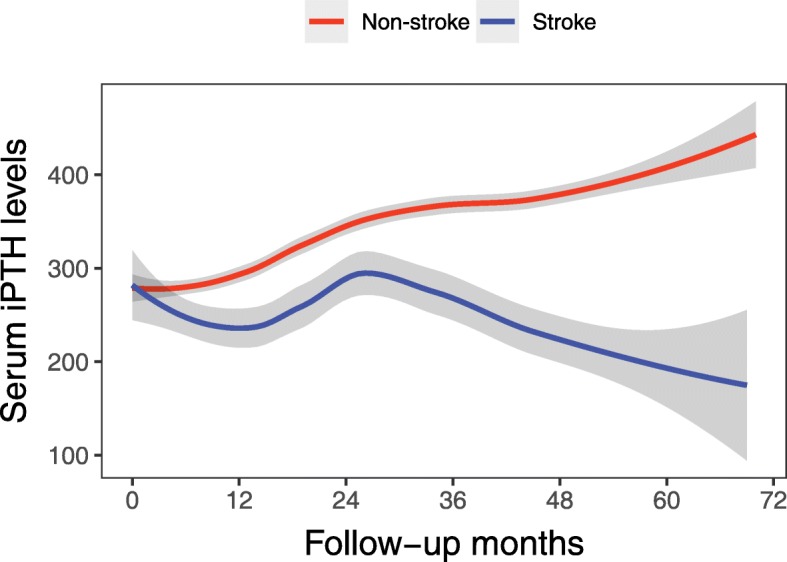
The difference in serum iPTH levels during follow-up between the stroke and non-stroke groups. The curves were fitted using the local polynomial regression line, and the gray region denotes the 95% confidence interval

The nonlinear regression curves displayed markedly different trends in iPTH levels during follow-up in the stroke group and the non-stroke group. The iPTH levels gradually decreased in the stroke group, but increased in the non-stroke group as the number of months of dialysis increased (Fig. 2).

### Relationship between baseline iPTH and stroke

Our data showed a significantly skewed distribution of baseline serum iPTH levels, and the probability density distribution was markedly different between the non-stroke group and the stroke group, with a significant leftward shift in the peak value for the stroke group, indicating that the serum iPTH levels were significantly lower in the stroke group (Supplemental Fig. 2a). Our nonlinear Cox regression analysis indicated a significant nonlinear correlation between baseline iPTH levels and the risk of stroke (*p*-value of the linear part = 0.2 and nonlinear part = 0.002). The curve of the relative stroke rate by baseline iPTH levels (referred to as 152 pg/ml) was J-shaped, suggesting that patients with low and markedly high levels of iPTH had a higher risk of stroke (Supplemental Fig. 2b).

**Fig. 3:**
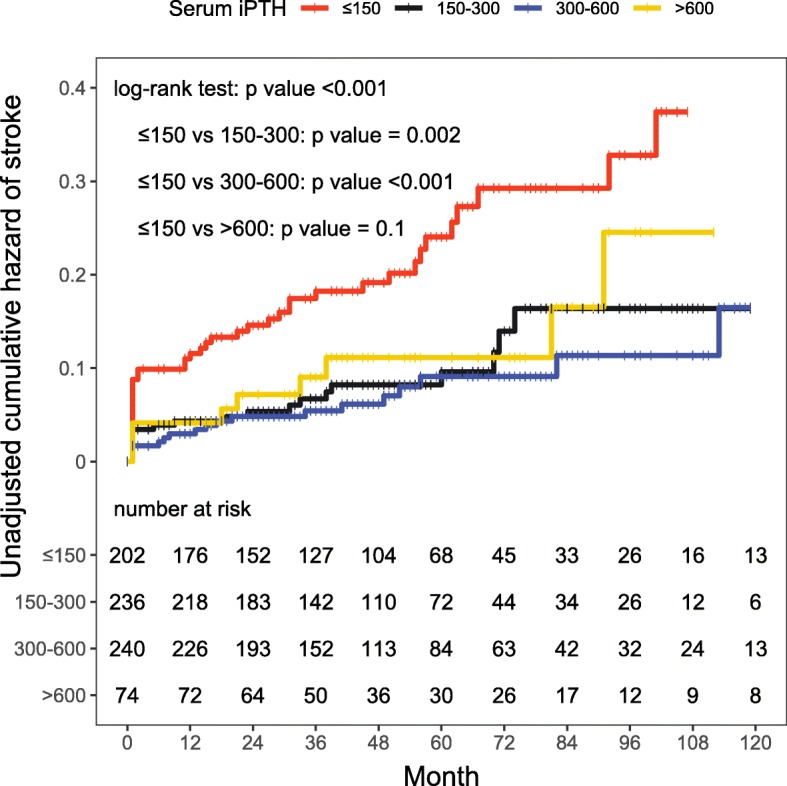
Kaplan-Meier analysis of stroke risk in groups stratified by different serum iPTH levels. Serum iPTH levels were divided into four groups: < 150, 150–300, 300–600, and > 600 pg/ml

A Kaplan-Meier analysis of stroke among patients with different baseline serum iPTH levels showed a significant difference in the cumulative risk of stroke between the groups (log-rank test, *p*-value < 0.001), and patients with low baseline iPTH levels (≤150 pg/ml) had an increased cumulative risk of stroke. The pairwise comparison between groups revealed significant differences between the ≤150 group and the 150–300 group and the 300–600 group (*p*-values = 0.002 and < 0.001, respectively), but significant differences were not observed between the ≤150 group and the > 600 group (p-value = 0.1, Fig. 3).

### Risk factors for stroke and composite endpoints

According to our univariate Cox regression analysis, increasing age and decreased DBP and iPTH levels combined with chronic heart disease, atrial fibrillation or diabetes, treatment with antiplatelet agents and not taking vitamin D supplements are common risk factors for stroke and composite endpoints (Table 2). Surprisingly, positive correlations were observed between the use of antiplatelet agents with the risk of stroke and composite endpoints, which we postulated might be a reverse epidemiological phenomenon, such as the treatment effect of statin on dialysis patients, or was attributed to the impact of the propensity treatment.

**Table 2:** Univariate Cox regression for stroke and composite endpoints

Variables	Model 1			Model 2	
	HR [95% CI]		***p***-value	HR [95% CI]	***p***-value
Age (years)	1.07 [1.05, 1.09]		< 0.001	1.05 [1.04, 1.06]	< 0.001
Gender (male)	0.72 [0.44, 1.18]		0.2	1.58 [1.13, 2.22]	0.008
Serum albumin (g/l)	0.96 [0.92, 1.00]		0.07	0.96 [0.94, 0.99]	0.005
Serum UA (mg/dl)	0.97 [0.86, 1.10]		0.6	0.98 [0.91, 1.07]	0.7
Hemoglobin (g/l)	1.00 [0.99, 1.01]		0.9	1.00 [0.99, 1.01]	0.5
Serum calcium (mmol/l)	1.44 [0.57, 3.63]		0.4	1.03 [0.57, 1.86]	0.9
Serum phosphorus (mmol/l)	0.55 [0.30, 1.03]		0.06	0.60 [0.40, 0.90]	0.01
iPTH levels					
≤ 150	Ref		–	Ref	–
150–300	0.43 [0.23, 0.81]		0.009	0.81 [0.54, 1.22]	0.3
300–600	0.38 [0.20, 0.72]		0.003	0.54 [0.35, 0.84]	0.006
> 600	0.58 [0.25, 1.33]		0.2	0.62 [0.34, 1.13]	0.1
SBP (mmHg)	1.00 [0.99, 1.01]		0.5	1.00 [0.99, 1.01]	0.9
DBP (mmHg)	0.97 [0.95, 0.98]		< 0.001	0.98 [0.97, 0.99]	< 0.001
Chronic heart disease (yes)	3.19 [1.95, 5.22]		< 0.001	3.04 [2.19, 4.23]	< 0.001
Atrial fibrillation (yes)	2.52 [1.09, 5.85]		0.03	2.88 [1.68, 4.92]	< 0.001
Diabetes (yes)	2.81 [1.72, 4.59]		< 0.001	2.43 [1.75, 3.37]	< 0.001
Vitamin D (yes)	0.38 [0.23, 0.63]		< 0.001	0.46 [0.32, 0.64]	< 0.001
Antiplatelet agents (yes)	3.33 [1.99, 5.58]		< 0.001	1.67 [1.20, 2.32]	0.002
Anticoagulants (yes)	0.72 [0.10, 5.22]		0.7	2.39 [1.12, 5.11]	0.02
Calcium agents (yes)	0.53 [0.31, 0.92]		0.02	0.68 [0.46, 1.00]	0.05
Statins (yes)	1.38 [0.80, 2.37]		0.3	0.82 [0.58, 1.14]	0.2

Model 1: Univariate model for stroke; Model 2: Univariate model for composite endpoints

According to our data, a significant inverse correlation between serum iPTH levels and age (Kendall’s rank correlation coefficient = − 0.15, *p*-value < 0.001) was observed. Then, an interaction term of serum iPTH levels and age was included in the multivariate Cox models of stroke and composite endpoints (Table 3). In the stroke model (Table 3, model 1), our results revealed a significant effect of the interaction between serum iPTH levels and age; furthermore, the baseline serum iPTH levels showed a more significant association with stroke than age. In contrast to the stroke model, a significant effect of the interaction between serum iPTH levels and age was not observed on the composite model (Table 3, model 2). Based on the plot of the effects of the interaction between serum iPTH levels and age in the stroke model (Supplemental Fig. 3), the preferred iPTH levels range from 150 and 300 pg/ml in younger patients (< 65 years) and 300 and 600 pg/ml in older patients (≤65 years).

**Table 3:** Multivariate Cox regression analysis of stroke and composite endpoints

Variables	Model 1		Model 2	
	HR [95% CI]	***p***-value	HR [95% CI]	***p***-value
Age (years)	1.03 [0.99, 1.06]	0.1	1.03 [1.01, 1.06]	0.008
Gender (male)			1.53 [1.04, 2.25]	0.03
Serum albumin (g/l)	0.99 [0.94, 1.05]	0.7	0.99 [0.95, 1.03]	0.6
Serum UA (mg/dl)	1.06 [0.91, 1.24]	0.4	0.99 [0.90, 1.08]	0.8
Serum calcium (mmol/l)	1.07 [0.31, 3.63]	0.9	1.13 [0.51, 2.52]	0.8
Serum phosphorus (mmol/l)	1.08 [0.52, 2.24]	0.8	1.11 [0.69, 1.81]	0.7
iPTH levels				
≤ 150	Ref	–	Ref	–
150–300	0.02 [0.00, 0.89]	0.04	0.48 [0.06, 3.59]	0.5
300–600	0.21 [0.01, 5.18]	0.3	0.22 [0.03, 1.77]	0.2
> 600	0.50 [0.00, 51.58]	0.8	0.33 [0.02, 5.29]	0.4
SBP (mmHg)	1.01 [1.00, 1.02]	0.06	1.00 [0.99, 1.01]	0.9
DBP (mmHg)	0.98 [0.96, 1.00]	0.04	1.00 [0.99, 1.02]	0.8
Chronic heart disease (yes)			2.34 [1.60, 3.43]	< 0.001
Atrial fibrillation (yes)	1.27 [0.48, 3.33]	0.6	1.16 [0.56, 2.40]	0.7
Diabetes (yes)	1.31 [0.73, 2.36]	0.4	1.53 [1.04, 2.26]	0.03
Vitamin D (yes)	0.42 [0.23, 0.76]	0.004	0.50 [0.33, 0.74]	0.001
Antiplatelet agents (yes)	1.72 [0.93, 3.15]	0.08	0.84 [0.57, 1.23]	0.4
Anticoagulants (yes)			0.94 [0.34, 2.63]	0.9
Calcium agents (yes)	0.49 [0.27, 0.89]	0.02	0.79 [0.51, 1.23]	0.3
Interaction term				
Age: iPTH < 150	Ref	–	Ref	–
Age: iPTH 150–300	1.06 [1.00, 1.13]	0.06	1.01 [0.98, 1.05]	0.5
Age: iPTH 300–600	1.02 [0.97, 1.07]	0.5	1.02 [0.98, 1.06]	0.3
Age: iPTH > 600	1.02 [0.93, 1.11]	0.7	1.03 [0.97, 1.08]	0.3

Variables with a *p*-value less than 0.1 in the univariate Cox regression analyses were selected to build the multivariate Cox regression models. Model 1: multivariate model for stroke; Model 2: multivariate model for composite endpoints

### Subgroup analysis for vitamin D supplementation

According to our multivariate Cox regression analysis, the consumption of a vitamin D supplement during follow-up was an independent protective factor both for stroke and the composite endpoints (model 1: HR, 0.42, 95% CI 0.24–0.74, *p*-value = 0.002; model 2: HR, 0.47, 95% CI 0.32–0.68, p-value < 0.001; Table 3). To further investigate the effects of vitamin D supplementation among different populations of CAPD patients, a subgroup analysis was performed. Regardless of the levels of serum calcium or phosphate, vitamin D supplementation was a significant protective factor for stroke. Interestingly, vitamin D supplementation was an independent predictive factor for stroke in male patients and older patients (HR 0.38, 95% CI 0.2–0.72, and HR 0.24, 95% CI 0.1–0.58, respectively). Additionally, vitamin D supplementation is associated with a reduced risk of stroke in patients with serum iPTH levels less than 600 pg/ml (Supplemental Fig. 4).

## Discussion

As studies have revealed, stroke is a serious complication associated with high rates of hospitalization, transfer to hemodialysis, and death [3, 26]. The median incidence of stroke was 18.9 per 1000 person-years in our cohort, which is markedly increased compared to the incidence among the general population of China (3.5 per 1000 person-years) [27], and a significantly increased proportion of composite endpoints among patients with stroke during follow-up. Furthermore, we noticed an interesting phenomenon in our cohort in which patients at the time of initiation and 5 years and 10 years after CAPD had a higher incidence of stroke. After these peaks, the incidence of stroke decreased gradually. Murray et al. found a peak incidence of stroke 1 to 2 months before and after initiation of hemodialysis or peritoneal dialysis, and the incidence of stroke decreased gradually during follow-up [28]. However, to our knowledge, the other two peaks over the CAPD period have not been described in previous studies. Based on our clinical practice, we presumed that these peaks may be attributed to the loss of residual renal function 5 years after CAPD and the gradually decreased peritoneal function at ten years.

Hyperparathyroidism is a common complication in patients with CAPD, and a modest increase in iPTH may represent an appropriate adaptive response to declining kidney function [22]. The target range of PTH levels for dialysis patients was suggested by the Kidney Disease Outcome Quality Initiative (K/DOQI) guidelines and the KDIGO guidelines based on studies of bone and mineral disorders in patients with CKD [22, 29]. Our study demonstrated that the relationship between baseline iPTH levels and the risk of stroke appeared to be J-shaped, indicating that low or markedly elevated baseline iPTH levels are associated with an increased risk of stroke. Furthermore, significantly lower iPTH levels were observed in the stroke group than in the non-stroke group, and a notable inverse correlation of iPTH levels was observed between stroke and non-stroke patients during follow-up. Low iPTH levels are also significantly correlated with vascular calcification, cardiovascular disease and mortality in dialysis patients [17, 18, 30, 31]. However, whether the decreased iPTH level is a cause or just a phenomenon associated with stroke in CAPD patients is still unclear.

Our study indicated a significant effect of the interaction between age and baseline iPTH levels on stroke. Importantly, in contrast to age, baseline iPTH levels were still a significant risk factor for stroke after adjusting for the interaction term and other confounders in the multivariate Cox regression model. Although age is a significant predictor of stroke [26, 32], the effect of the interaction between age and iPTH levels should not be ignored. Morrone LF et al. [23] reported a significant effect of age on the interaction effect of iPTH levels and the Charlson Index as a predictor of mortality in patients with ESRD who were receiving dialysis. Furthermore, preferred iPTH levels were observed for different age groups that reduced the risk of stroke. Thus, individual therapies for iPTH may be reasonable at different age groups.

Vitamin D supplementation is widely used to treat vitamin D deficiency or insufficiency, secondary hyperparathyroidism, and hypocalcemia in CAPD patients. However, treatment with vitamin D supplements in dialysis patients is still controversial. In our study, regardless of any strategies of vitamin D supplementation during follow-up, vitamin D supplementation was an independent predictive factor for stroke in our CAPD patients, and the predictive effects were more significant in male and younger patients and even in patients with lower iPTH levels. Vitamin D supplementation has been shown to decrease the activity of the RAAS, decrease inflammation, and improve endothelial function, and its deficiency is significantly associated with stroke [11]. However, the benefit of vitamin D supplementation for cardiovascular disease has not been demonstrated [33]. Thus, individualized treatment with vitamin D supplements may be more important for CAPD patients due to the complex dynamic equilibrium of the calcium-parathyroid hormone-vitamin D axis.

However, our results should be interpreted cautiously. First, a key limitation is that we conducted a retrospective study of a single Chinese center, and the confounding effects of the indication for vitamin D administration as a propensity treatment or the consumption of nutritional vitamin D on our cohort should not be ignored. These findings should be validated in different centers. Second, a high proportion of patients withdrew from the study. Third, approximately 3% of included patients died of unknown causes at home, and some of the deaths could have been attributed to stroke but were not counted in the stroke group, which may lead to an underestimation of the incidence of stroke.

## Conclusions

CAPD patients suffered a high risk of stroke, especially at the initiation and 5 years and ten years after CAPD. Lower iPTH levels were significantly associated with an increased risk of stroke, particularly a lower baseline iPTH level, which was an early independent predictor for stroke, but vitamin D supplementation may reduce the risk of stroke in patients with CAPD. Furthermore, a significant effect of the interaction between age and iPTH levels on predicting the stroke risk was observed. Consistent with other studies, the weak correlations of stroke with iPTH levels and vitamin D supplementation should be interpreted with caution. However, individualized therapy would be rational for CAPD patients.

## Supplementary information

**Additional file 1 Supplemental Table 1**. Comparison of serum iPTH levels during follow-up between the stroke and nonstroke groups.

**Additional file 2.**

**Additional file 3.**

**Additional file 4.**

**Additional file 5.**

## Data Availability

The datasets generated and/or analyzed during the current study are available from the corresponding author upon reasonable request.

## Abbreviations

CAPD: Continuous ambulatory peritoneal dialysis
ESRD: End-stage renal disease
CKD-MBD: Chronic kidney disease-mineral and bone disorder
CVD: Cardiovascular disease
SD: Standard deviation
IQR: Interquartile range
HR: Hazard ratio
CI: Confidence interval
MAD: Median absolute deviation
PTH: Parathyroid hormone
iPTH: Intact parathyroid hormone
SBP: Systolic blood pressure
DBP: Diastolic blood pressure
MAP: Mean arterial pressure
UA: Uric acid
CCBs: Calcium channel blockers
RAAS: Renin-angiotensin-aldosterone system
KDIGO: Kidney Disease Improving Global Outcomes
K/DOQI: Kidney Disease Outcome Quality Initiative
