# Supplementary material for: Effects of parathyroid hormone and vitamin D supplementation on stroke among patients receiving peritoneal dialysis
Source: BMC Nephrol. 2020 May 18;21:183. doi: 10.1186/s12882-020-01817-6 (PMC7236177; doi:10.1186/s12882-020-01817-6)
Supplement: Supplementary file 1 — Additional file 1 Supplemental Table 1. Comparison of serum iPTH levels during follow-up between the stroke and nonstroke groups. [file 12882_2020_1817_MOESM1_ESM.docx]

Supplemental Table 1. Comparison of serum iPTH levels during follow-up between the stroke and nonstroke groups.

| **Characteristics** | **Non-stroke** | **Stroke** | **p** |
| --- | --- | --- | --- |
| iPTH (median [IQR]) | 244.2 [169.6, 369.1] | 188.8 [105.8, 286.2] | 0.001 |
| log iPTH (median [IQR]) | 5.4 [5.1, 5.8] | 5.2 [4.4, 5.6] | <0.001 |
| Time-averaged iPTH (median [IQR]) | 268.3 [184.9, 388.6] | 210.8 [108.1, 324.0] | 0.001 |
| MAD of iPTH (median [IQR]) | 66.2 [41.7, 106.5] | 52.9 [34.4, 93.2] | 0.032 |
