# Supplementary figures and images for: Effects of parathyroid hormone and vitamin D supplementation on stroke among patients receiving peritoneal dialysis
Source: BMC Nephrol. 2020 May 18;21:183. doi: 10.1186/s12882-020-01817-6 (PMC7236177; doi:10.1186/s12882-020-01817-6)

Incidence of stroke per year (%)

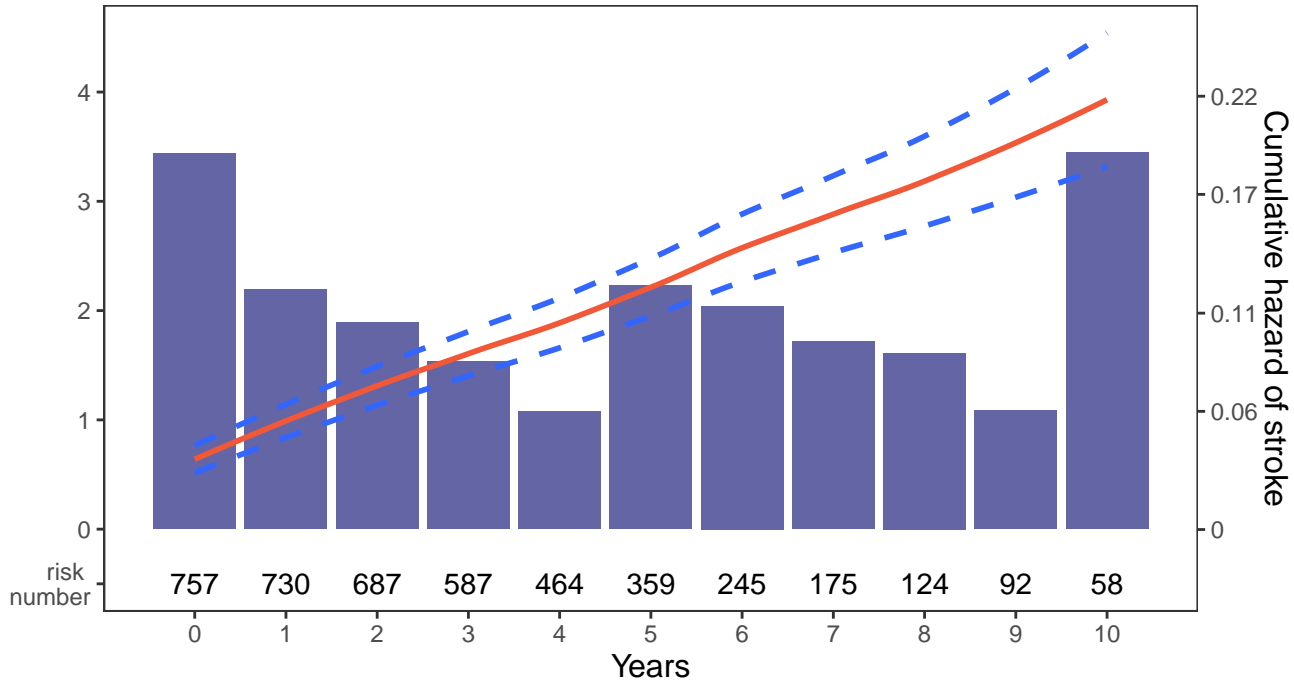

Supplement: Supplementary file 2 — Additional file 2. [file 12882_2020_1817_MOESM2_ESM.pdf]

**a**

Non-stroke    Stroke

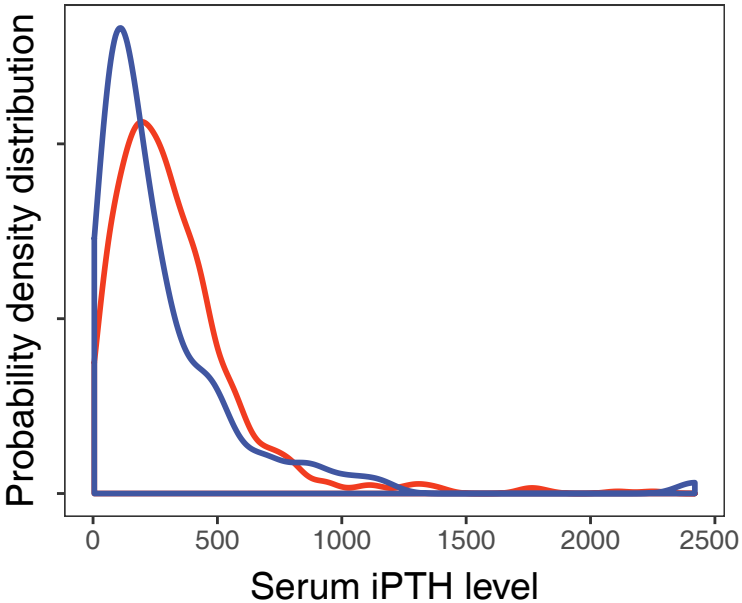

**b**

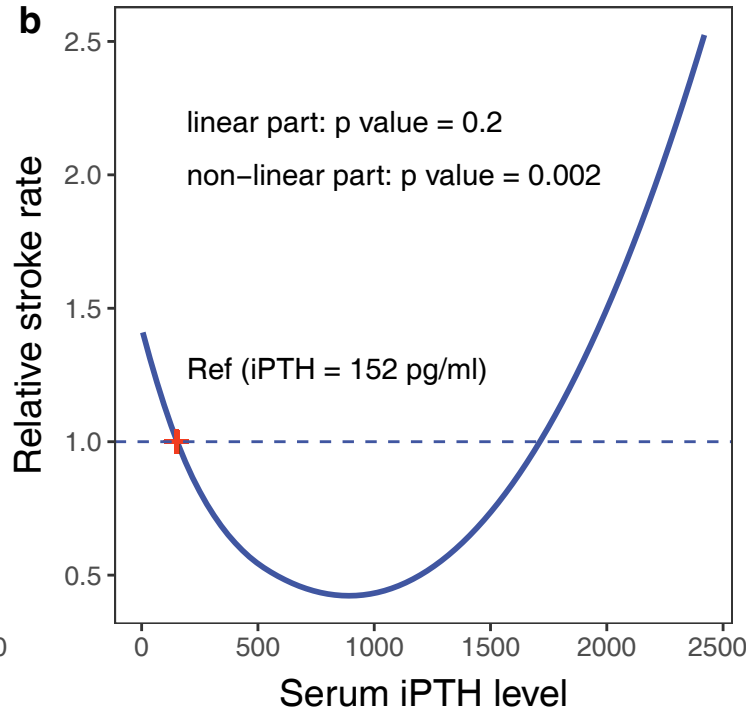

Supplement: Supplementary file 3 — Additional file 3. [file 12882_2020_1817_MOESM3_ESM.pdf]

iPTH levels    —  $\leq 150$     — 150-300    — 300-600    —  $>600$

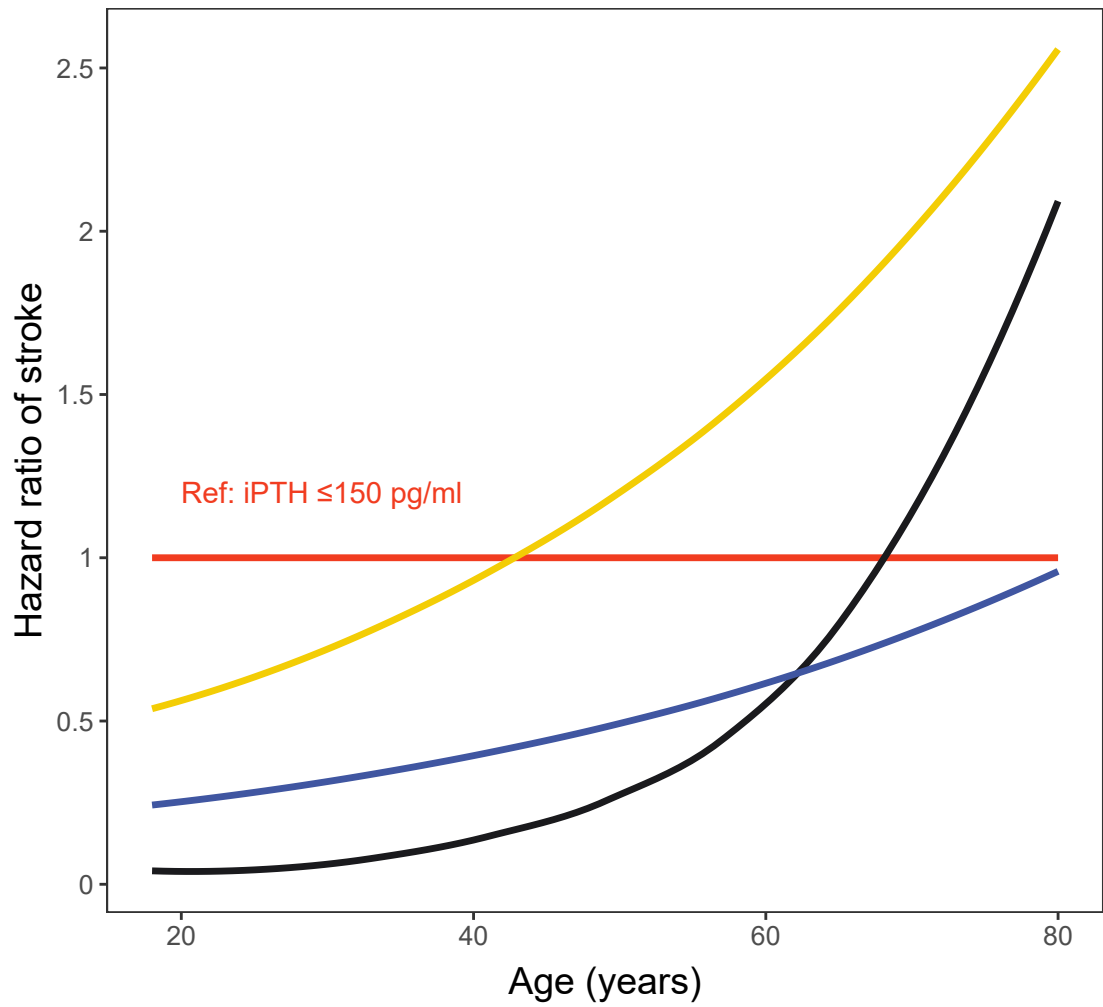

Supplement: Supplementary file 4 — Additional file 4. [file 12882_2020_1817_MOESM4_ESM.pdf]

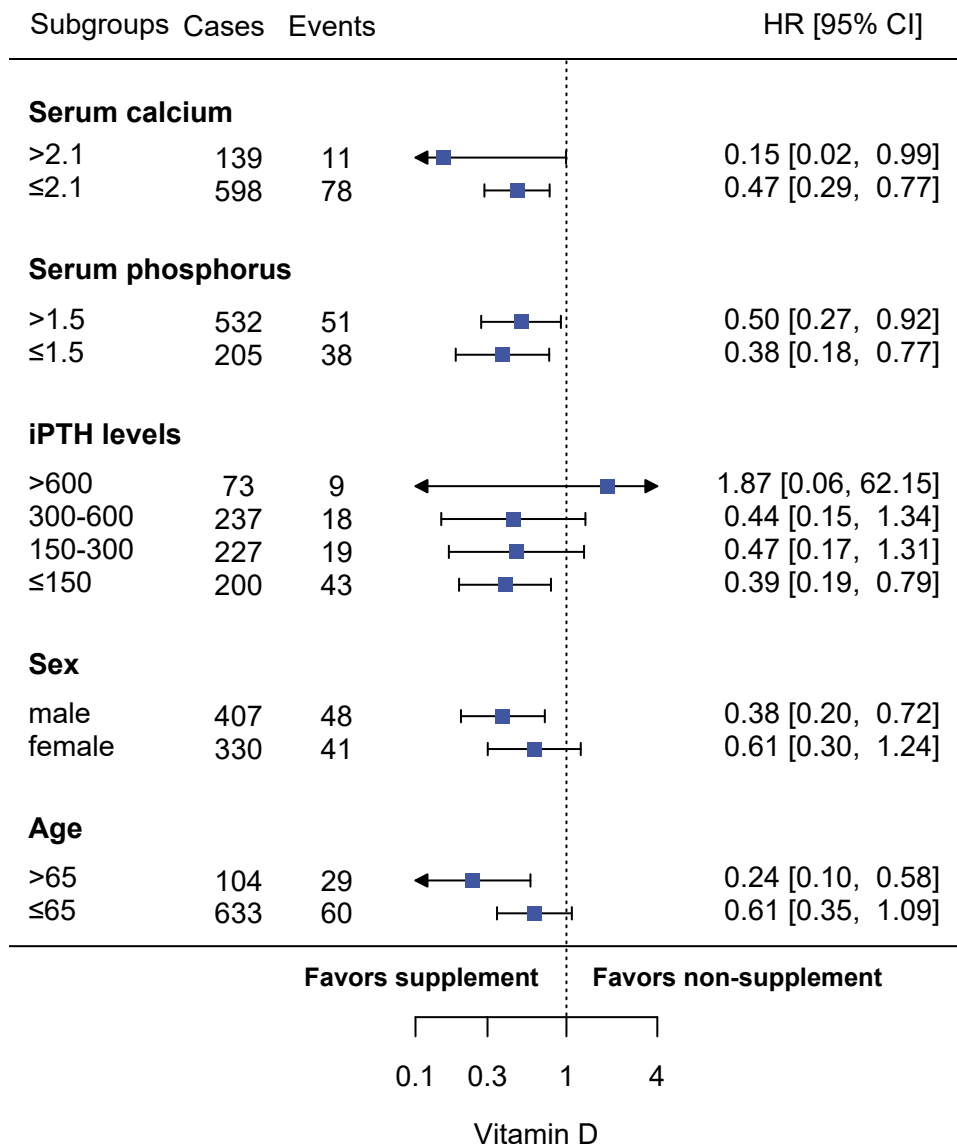

Supplement: Supplementary file 5 — Additional file 5. [file 12882_2020_1817_MOESM5_ESM.pdf]
